# Delivery of continuously-varying stimuli using channelrhodopsin-2

**DOI:** 10.3389/fncir.2013.00184

**Published:** 2013-12-06

**Authors:** Tatjana Tchumatchenko, Jonathan P. Newman, Ming-fai Fong, Steve M. Potter

**Affiliations:** ^1^Max Planck Institute for Brain ResearchFrankfurt am Main, Germany; ^2^Department of Biomedical Engineering, Georgia Institute of TechnologyAtlanta, GA, USA; ^3^Department of Physiology, Emory University School of MedicineAtlanta, GA, USA

**Keywords:** channelrhodopsin-2, linear response theory, dynamical systems, neural circuits, networks and dynamical systems, circuit dynamics, optogenetics, electrophysiology methods

## Abstract

To study sensory processing, stimuli are delivered to the sensory organs of animals and evoked neural activity is recorded downstream. However, noise and uncontrolled modulatory input can interfere with repeatable delivery of sensory stimuli to higher brain regions. Here we show how channelrhodopsin-2 (ChR2) can be used to deliver continuous, subthreshold, time-varying currents to neurons at any point along the sensory-motor pathway. To do this, we first deduce the frequency response function of ChR2 using a Markov model of channel kinetics. We then confirm ChR2's frequency response characteristics using continuously-varying optical stimulation of neurons that express one of three ChR2 variants. We find that wild-type ChR2 and the E123T/H134R mutant (“ChETA”) can pass continuously-varying subthreshold stimuli with frequencies up to ~70 Hz. Additionally, we find that wild-type ChR2 exhibits a strong resonance at ~6–10 Hz. Together, these results indicate that ChR2-derived optogenetic tools are useful for delivering highly repeatable artificial stimuli that mimic *in vivo* synaptic bombardment.

## Introduction

The network response to continuously-varying stimuli is at the core of cognitive and sensory processing. To understand how neuronal networks encode and process continuously-varying input, a sensory organ is presented with a precisely-defined stimulus and evoked spiking activity is recorded from a corresponding brain region. The power of this technique for deducing network encoding properties has been demonstrated in numerous preparations, including the retina, (Warland et al., 1997; Chichilnisky, 2001), antennal and mechanosensory systems of insects (Warland et al., 1992; Geffen et al., 2009), and somatosenory, auditory, and visual systems of mammals (Arabzadeh et al., 2003; Lesica et al., 2007; Kayser et al., 2010). However, as stimuli delivered to sensory organs propagate to higher brain areas, intrinsic noise and modulatory input from secondary brain regions can interfere with controlled input signals. For studies concerning the function of neural circuits that are several synapses removed from sensory input, the direct introduction of continuously-varying currents to a neural population may provide a more straightforward way to deduce circuit response dynamics.

Optogenetic methods allow precise control of spike times using brief light pulses to excite light-gated ion channels and pumps, such as channelrhodopsin-2 (ChR2) (Boyden et al., 2005; Gunaydin et al., 2010; Mattis et al., 2011). Pulsed optical stimulation using ChR2 dictates a spiking response that is tightly locked to each stimulus by briefly overriding neuronal dynamics. This stands in contrast to the highly variable, sub-threshold currents recorded from cortical neurons during natural sensory processing *in vivo* (Destexhe et al., 2003). We hypothesized that using relatively low intensity, continuously modulated optical stimuli to excite ChR2 might allow conductance fluctuations that mimic *in vivo*-like synaptic bombardment and leave the decision of when and how often to spike to individual cells (Mainen and Sejnowski, 1995; Tchumatchenko et al., 2011). Surprisingly, while the response properties of microbial opsins to optical pulses have been studied extensively (Mattis et al., 2011), little is known about their ability to relay fluctuating light signals.

In order for ChR2 to be useful for delivering continuously-varying currents, it must allow (1) sufficient bandwidth to mimic synaptic communication and (2) repeatable current waveforms to be delivered across trials. Here, we address these requirements theoretically and experimentally. We find that wild-type ChR2 (ChR2) (Boyden et al., 2005) supports significant photocurrents up to 69 Hz, the H134R mutant (ChR2_R_) (Nagel et al., 2005) up to 37 Hz, and E123T/H134R mutant (ChR2_A_; also known as “ChETA”) (Gunaydin et al., 2010) up to 73 Hz, and show that evoked current waveforms are extremely repeatable across trials. Using the model, we find that the bandwidth over which ChR2_R_ and ChR2 can convey time-varying stimuli is reduced with increasing membrane potential but that ChR2_A_'s passband is unaffected. Finally, we show that wild type ChR2 supports a strong resonance with a natural frequency around 10 Hz. This resonance is present, but significantly attenuated, in the H134R and E123T/H134R mutants.

## Results

### ChR2's frequency response

**Figure 1:**
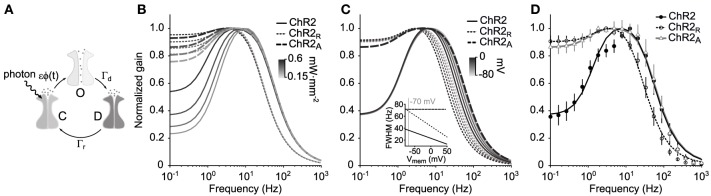
**ChR2's amplitude response function. (A)** Illustration of the three-state Markov channel model described by Equations 1–3. The transition rates between open, *O*, desensitized, *D*, and closed, *C*, states are ϵϕ(*t*), Γ_*r*_, and Γ_*d*_, respectively. **(B)** Amplitude response functions |*F*_ChR2_(2π*f*)| for the model are shown for three ChR2 variants using different mean illumination intensities (0.15–0.6 mW·mm^−2^) and parameters in Table 1. **(C)** Voltage dependence of ChR2's amplitude response function. ChR2 and ChR2_R_ both have a voltage-dependent desensitization rate, Γ_*d*_(*v*), which results in decreased bandwidth as the membrane potential increases. ChR2_A_ does not have a voltage dependent desensitization rate and therefore has a stable bandwidth across membrane potentials. **(D)** Predicted amplitude response of each ChR2 variant compared to the experimentally measured response for a mean illumination intensity of 0.35 mW·mm^−2^. Error bars are ±1 STD.

In this study, we sought a general description of ChR2's dynamics that captured the ability of both ChR2 and its engineered variants to convey continuously-varying stimuli. To do this, we determined the frequency response function of a population of channels expressed by a single cell, *F*_ChR2_(ω), using a three-state Markov model of ChR2's channel kinetics (Figure 1A) (Nagel et al., 2003). The rate equations governing the model's state transitions are

(1) $$\dot{O}(t)=\epsilon \phi (t)C(t)-\Gamma_{d}O(t)$$

(2) $$\dot{D}(t)=\Gamma_{d}O(t)-\Gamma_{r}D(t)$$

(3) C(t)=1−O(t)−D(t),

where the state variables *O*, *D*, and *C* are the probabilities of a channel being open, desensitized, or closed, respectively. Γ_*d*_ and Γ_*r*_ are the rates of channel desensitization and recovery. ϵ is the quantum efficiency of ChR2 and ϕ(*t*) is the instantaneous photon flux (light intensity) impinging on a single channel. ϕ(*t*) can be modulated by changing the light intensity of a stimulating light source as a function of time.

The conductance of ChR2 across the cell membrane is proportional to the number of channels that occupy the open state. Therefore, *F*_ChR2_(ω) can be thought of as a frequency- and phase-dependent transition rate from the channels' closed to open states in response to a continuously-varying stimulus. Since individual channels switch between states discretely, *F*_ChR2_(ω) describes the transformation of arbitrary optical waveforms to intracellular current under the assumption that a large number of channels are present in the cell's membrane. *F*_ChR2_(ω) is given by

(4) $$F_{\text{ChR2}}(\omega )=\frac{C_{0}\text{​}\left(j\omega +\Gamma_{r}\right)}{-\omega^{2}+j\omega \text{​}\left(\Gamma_{r}+\epsilon \phi_{0}+\Gamma_{d}\right)+\epsilon \phi_{0}\Gamma_{r}+\epsilon \phi_{0}\Gamma_{d}+\Gamma_{r}\Gamma_{d}}.$$

A detailed derivation of *F*_ChR2_(ω) can be found in the Methods section.

The amplitude response function, |*F*_ChR2_(ω)| (Equations 4 and 19), is the component of *F*_ChR2_(ω) that indicates the frequency-dependent gain of the channel population in response to fluctuating light signals. The predicted amplitude response functions for ChR2, |*F*_ChR2_(ω)|, ChR2_R_, |*F*_ChR2_R__(ω)|, and ChR2_A_,|*F*_ChR2_A__(ω)|, are shown in Figure 1B for different mean illumination intensities. Model parameters were obtained for each ChR2 variant by fitting the predicted amplitude response function (Equation 19) to the experimental estimate (Table 1; Methods section “Delivering time varying currents using ChR2”).

**Table 1:** **Markov model parameters for each ChR2 variant obtained from fitting the amplitude response function (Equation 19) to the empirically derived response function**.

**Variant**	**ϵϕ_0_ (s^−1^)**	**Γ_*d*_ (s^−1^)**	**Γ_*r*_ (s^−1^)**
ChR2	6.51	236.35	3.60
ChR2_R_	1.16	126.74	8.38
ChR2_A_	0.96	254.63	5.57

|*F*_ChR2_(ω)| has a high frequency cutoff (width at half maximum) of 69 Hz. It should be noted that this cutoff value is defined relative to ChR2's peak conductance, and not in terms of absolute photocurrents. For this reason, it is still possible to use ChR2 to deliver physiologically significant photocurrents at stimulation frequencies exceeding 69 Hz. The shape of |*F*_ChR2_(ω)| indicates that ChR2 exhibits a significant resonance with a natural frequency around 6–10 Hz. This feature explains the large peak to steady-state (DC) ratio of ChR2-mediated photocurrents in response to pulsed stimuli (Gunaydin et al., 2010; Mattis et al., 2011).

In agreement with previous characterizations, ChR2_R_ is significantly slower than ChR2 and |*F*_ChR2_R__ (ω)| has a cutoff frequency at 37 Hz (Mattis et al., 2011). While ChR2_R_ supports a resonance in the 3–4 Hz range, its effect is significantly reduced compared to ChR2. The bandwidth of ChR2_A_ was similar to that of ChR2 with a high frequency cutoff of 73 Hz. |*F*_ChR2_A__ (ω)| displayed a moderate resonance that peaked at 3–5 Hz depending on the mean light intensity of the stimulation waveform.

### Voltage dependence of channel kinetics

For some ChR2 variants, channel kinetics are dependent on the membrane potential. The off time of ChR2- and ChR2_R_-evoked currents grow with increasing membrane potentials (Mattis et al., 2011). Additionally, the time-to-peak conductance for ChR2 increases approximately linearly with membrane potential (Berndt et al., 2011; Mattis et al., 2011). ChR2_A_ does not have voltage dependent kinetics (Berndt et al., 2011; Mattis et al., 2011). Therefore, for the ChR2 and ChR2_R_ variants, the transition rates Γ_*r*_ and Γ_*d*_ in the Markov model (Equations 1–3) both could be voltage dependent. To understand how voltage-dependent kinetics affect the bandwidth of each ChR2 variant, we derived the transient response of our model to a delta pulse of magnitude ϕ_0_ and to a downward step to zero from initial intensity ϕ_0_. The response to a delta light pulse, (“on-dynamics”) is given by

(5) $$O^{\text{on}}(t)=\epsilon \phi_{0}\exp\text{​}\left(-\Gamma_{d}t\right)\theta (t)$$

where θ(*t*) is the Heaviside theta function. The response to a downward step (“off-dynamics”) is given by

(6) $$O^{\text{off}}(t)=\exp\text{​}\left(-\Gamma_{d}t\right)\theta (t)\frac{\Gamma_{r}\epsilon \phi_{0}}{\Gamma_{d}\Gamma_{r}+\Gamma_{d}\epsilon \phi_{0}+\Gamma_{r}\epsilon \phi_{0}}.$$

The long time dynamics of both *O*^on^ and *O*^off^ are dominated by Γ_*d*_ when using biophysically relevant parameters. Therefore, to capture the effect of voltage on channel kinetics, we assumed a linear relationship between the voltage and Γ_*d*_ according to Γ_*d*_(*v*) = Γ_*d*_(1 − 0.0056(*v* + 70 mV)) (Mattis et al., 2011). We then recalculated the amplitude response function at membrane potentials ranging from −80 to 0 mV (Figure 1C). Increases in membrane potential affected the high-frequency cutoff for the ChR2 and ChR2_R_ and had a large effect on channel bandwidth. As the membrane voltage increased from −80 to 0 mV, the bandwidth of the amplitude response function decreased by 37% for both variants (Figure 1C, inset). In contrast, ChR2_A_'s constant bandwidth across voltages makes it well suited for introducing continuously-varying conductances into cells that are not voltage-clamped.

### Robustness of the frequency response function

The linear time-invariant frequency response function has the greatest predictive power for stimuli with low peak-to-peak amplitudes. To test the robustness of the frequency response function when using larger amplitude inputs, we compared it with the complete response arising from time-varying light amplitude. Sinusoidal inputs with mean intensity of ϕ_0_ = 0.35 mW·mm^−2^ and different amplitudes δϕ were used to drive the linear time-invariant frequency response (Equation 4) according to

(7) $$O(t)=O_{0}+\epsilon \delta \phi F_{\text{ChR2}}(\omega )\exp(j\omega t)$$

The complete response dynamics of the Markov model were obtained by inserting the offset and amplitude parameters, ϕ_0_ and δϕ, into the sinusoidal drive (Equation 8) and numerically integrating the corresponding differential Equations (1–3) to obtain *O*(*t*).

**Figure 2:**
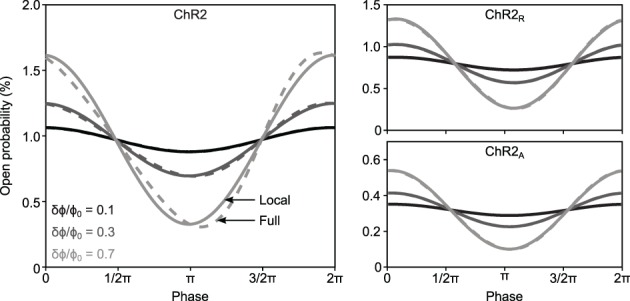
***F*_ChR2_(ω)'s linear time-invariant response versus complete model dynamics for different ChR2 variants**. The time-invariant *F*_ChR2_(ω) approximation (solid lines) and the complete dynamics (dashed lines) of *O*(*t*) are shown in response to 5 Hz sinusoidal stimuli for ChR2 (left), ChR2_R_ (right, top) and ChR2_A_ (right, bottom). Gray shades represent different sinusoidal amplitudes normalized to the mean stimulus intensity, δϕ/ϕ_0_ = 0.1, 0.3, and 0.7.

The response predicted by *F*_ChR2_(ω) and complete solutions will match when |*i*ω + ϵϕ_0_| ≫ ϵδϕ. This condition guarantees that terms proportional to exp(*j*ω*t*) dominate over higher order terms in the derivation of the *F*_ChR2_(ω) response function (Equations 13, 14). This condition is fulfilled when δϕ ≪ ϕ_0_ or for frequencies ω ≫ ϵϕ_0_. We compared simulated and time-invariant solutions to Equations 1–3 and found that the frequency response function provided a good approximation of the complete dynamics, even for relatively large stimulus amplitudes and low stimulus frequencies, where deviations between the linear time-invariant response and complete model dynamics are largest (5 Hz input; Figure 2). The deviation between *F*_ChR2_(ω) and complete dynamics was negligible for higher stimulus frequencies (20 Hz input; Supplementary Figure S4).

### Delivering continuously-varying currents using ChR2

**Figure 3:**
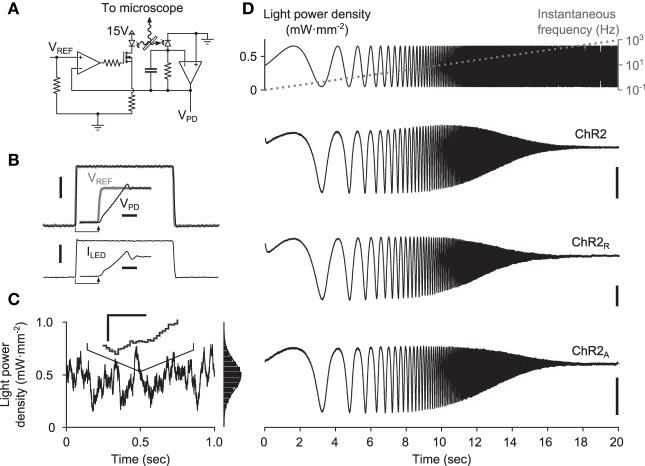
**Delivery of continuously-varying stimuli to neurons using ChR2. (A)** Simplified schematic of the LED driver in optical feedback mode. The circuit uses an amplified photodiode to compensate for the non-linearities and temperature dependence of the LED, allowing arbitrary waveforms to be delivered to cells. **(B)** A 1-ms LED pulse, V_PD_ (black), versus the reference voltage, V_REF_ (gray). The current sourced to the LED is shown in the lower plot. Scale bars, 1 mW·mm^−2^ (top) and 250 mA (bottom). Insets show the zoomed step onset with corresponding 5 μs scale bars. **(C)** A computer generated Gaussian stimulus (gray) signal and the recorded light waveform (black). The lines overlap almost perfectly, making the reference voltage (gray) difficult to see. An inset shows a zoomed portion of the sequence. Scale bars, 0.05 mW·mm^−2^ and 500 μs. An amplitude histogram of the sequence, with a best-fit Gaussian distribution, is shown to the right. **(D)** Responses to frequency chirp stimuli for each ChR2 variant tested. The top plot shows the stimulus waveform (black) along with the instantaneous frequency profile (gray) and bottom plots show evoked current waveforms. Scale bars, 100 pA.

To verify *F*_ChR2_(ω) experimentally, cultured cortical cells expressing either ChR2, ChR2_R_, or ChR2_A_ were stimulated with spatially uniform blue light (465 nm at peak intensity) using a light emitting diode (LED), while somatic photocurrents were recorded using whole-cell patch clamp (Methods). To ensure that optical stimuli tracked intended stimulus waveforms, we developed a custom LED driver that used optical feedback to compensate for the static non-linearities and temperature dependence associated with the LED light source (Figures 3A–C; Methods).

To gain an initial confirmation of each variant's ability to relay continuously-varying photocurrents, we stimulated cells with swept frequency cosine (“chirp”) stimuli (Figure 3D; Methods). Chirp inputs allow time and frequency characteristics of each variant to be read directly from the photocurrent time-series. Each variant displayed a characteristic decay in evoked current amplitude with increasing frequency, consistent with the model prediction. Additionally, the slightly increased midband amplitude of ChR2-evoked photocurrents provided indications of a bandpass effect.

Because the full dynamics of ChR2 are time-variant, the estimated frequency response (Equation 22; Methods) will vary depending on the stimulus choice. Our goal was to use ChR2 to deliver stimuli that mimic *in vivo*-like synaptic bombardment. Therefore, we used optical stimuli consisting of 10-s realizations of a filtered Gaussian noise signal (time constant: τ_*s*_ = 50 ms, mean ± standard deviation: μ_*s*_ = 0.4 ± σ_*s*_ = 0.08 mW·mm^−2^; Methods). We chose stimuli with these parameters because they evoked membrane voltage waveforms with similar amplitude and frequency characteristics to those obtained from *in vivo* recordings of sensory cortical neurons in the high-conductance state (Supplementary Figure S1) (Destexhe et al., 2003).

We measured the empirical frequency response function to the Gaussian noise stimulus, $\hat{F}$_ChR2_(ω), of cells expressing ChR2 (*n* = 9 cells), ChR2_R_ (*n* = 4 cells), or ChR2_A_ (*n* = 6 cells) (Methods; Equations 21–24). We then compared the empirical amplitude responses for each variant, |$\hat{F}$_ChR2_(ω)|, with their theoretical counterparts. We observed good agreement between the empirically derived and predicted amplitude response functions, although some differences exist (Figure 1D). For instance, both |$\hat{F}$_ChR2_R__(ω)| and |$\hat{F}$_ChR2_(ω)| have small downward deviations from the predicted response at ~5 Hz, which is more pronounced for ChR2. Additionally, the predicted frequency response tends to slightly overestimate the measured gain at frequencies above 100 Hz. Because stimuli were spatially homogeneous and applied over the extent of the dentritic arbor, the lower-than-predicted response to high frequency stimuli may result from passive dendritic filtering of evoked currents.

To examine how the choice of stimulus waveform might change the channels' response properties, we recalculated the amplitude response function using photocurrents recorded in response to chirp stimuli (Supplementary Figure S2). Since chirp stimuli are sinusoidal, they result in a U-shaped amplitude distribution that emphasizes extreme stimulus amplitudes (0.05 and 0.65 mW·mm^−2^; Supplementary Figure S3). Because of large sinusoidal amplitude relative to the steady state light level (δϕ/ϕ_0_ ≈ 1) and the overabundance of extreme values, chirp stimuli were less capable of meeting the condition |(*i*ω + ϵϕ_0_)| ≫ ϵδϕ, which ensures agreement between the response predicted by *F*_ChR2_(ω) and complete model solutions. Consequently, the amplitude response function estimated from chirp inputs deviates from the analytical amplitude response (Equation 19). As predicted using the model, these deviations primarily affect low frequencies *f* ≤ 5Hz and are most prominent in wild-type ChR2 (Methods section “Robustness of the frequency response function”; Figure 2).

Despite these imperfections, both our theoretical and our empirical results indicate that all three channel types are capable of transmitting fluctuating current stimuli to populations of cells in a physiologically relevant frequency range (up to ~100 Hz). Furthermore, because the model provides a tractable description of channel dynamics, it serves as a useful tool for predicting the bandwidth and resonance of new channels based on measurable physiological parameters.

### Reliability of continuously-varying ChR2-evoked currents

**Figure 4:**
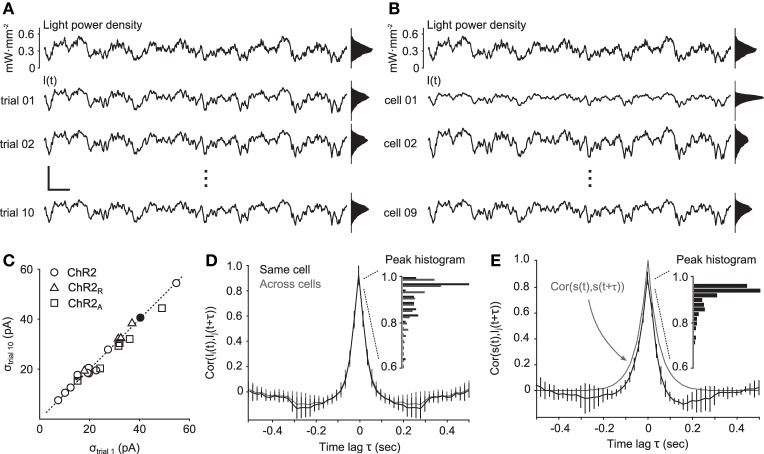
**Reliability of continuously-varying neuronal photostimulation. (A)** Intracellular currents from a single cell during Gaussian stimuli. The top trace is a portion of a 10-s Gaussian stimulus sequence. The bottom three traces show the intracellular currents recorded during different presentations of the same stimulus waveform. Scale bars, 200 pA and 200 ms. Scale bars apply to all time series traces in the figure. **(B)** The same stimulus waveform used in **(A)**, and the corresponding evoked responses from different cells. **(C)** The standard deviation of the photocurrent induced on the first trial of stimulation versus on the last trial. The dotted line is identity. Points near the identity line indicate that there was little or no decrease in stimulus efficacy across trials. The filled dot corresponds to the cell in **(A)**. **(D)** Normalized cross-correlation functions of photocurrents between neurons (gray) or autocorrelation function of photocurrents within the same neuron (black). The inset shows a histogram of peak correlation coefficients. **(E)** Normalized cross-correlation function between the stimulation process *s*(*t*) and recorded photocurrents. The gray line is the autocorrelation function of the stimulation process. The inset shows a histogram of peak correlation coefficients. Unless otherwise noted, data in this figure were obtained from cells expressing ChR2.

In order for continuously-varying photostimulation to be useful in experimental settings, evoked current waveforms must be highly repeatable. Therefore, we measured the reliability of photocurrent waveforms across trials. As expected, evoked photocurrents looked like smoothed versions of the stimulus signal due to the low-pass effect of ChR2's amplitude response function (Figure 4). Evoked current waveforms were remarkably stable across trials. There there was no systematic change in the amplitude of evoked currents during repeated applications of a stimulus waveform (Figures 4A,C).

We next examined the distribution of evoked current amplitudes across cells (Figure 4C). The average standard deviation of photocurrents evoked by ChR2 was 26.7 pA. ChR2_R_ and ChR2_A_ delivered only slightly larger current fluctuations than ChR2, with a mean standard deviations of 32.0 pA and 31.4 pA, respectively. The similarity in evoked current amplitudes between ChR2 and the engineered variants is likely due to ChR2's resonance, which makes the channel most sensitive to fluctuating stimuli with power in the 10 Hz range, as opposed to steady state inputs.

Finally, to determine the reliability of evoked currents across cells, we calculated the normalized cross-correlation function, *c*_*s*_, *I*_*i*_, between the light power density, *s*(*t*) and photocurrents *I*_*i*_(*t*) for each cell, *i*, and across cells, *c*_*I*_*i*_, *I*_*j*__. For ChR2, the median peak value of *c*_*I*_*i*_, *I*_*j*__ was 0.96, indicating strong correlations between evoked currents in different cells (Figure 4D). The median peak value of *c*_*s*, *I*_*i*__ was 0.92, indicating strong correlations between evoked currents and the stimulus waveform (Figure 4E). Additionally, the similarity in shape between *c*_*I*_*i*_, *I*_*j*__ and the autocorrelation of the stimulation process, *c*_*s*, *s*_, indicates that temporal features of the stimulus were accurately converted into photocurrents, as predicted by the passband of the frequency response function (Figure 1).

## Discussion

Optogenetic methods offer genetic specificity, elimination of electrical recording artifacts, and increasingly specialized functionality (Berndt et al., 2011; Mattis et al., 2011). Because of these advantages, optogenetic methods are often used for direct manipulation of neuronal subpopulations in order to understand their function (Cardin et al., 2009; Sohal et al., 2009). Typically, pulsed optical stimuli are used to elicit neural responses. However, for studies that seek to understand information transmission in neural circuits, continuously modulated photocurrents that mimic synaptic bombardment offer several advantages over pulsed stimuli. Continuously modulated photocurrents provide highly controlled subthreshold inputs, while leaving the decision of when and how often to spike to individual neurons. Therefore, the spikes recorded from the network response to such continuously modulated photocurrents carry the signatures of innate spike generation mechanisms as well as those of interneuronal connectivity and thereby offer the possibility of revealing underlying network encoding strategies.

Previous studies of ChR2's response function have provided mixed results for understanding the channel's ability to relay time varying input. A preliminary abstract on the ChIEF (Lin et al., 2009) variant's response dynamics in HEK cells described a low-pass rather than a band-pass response (Neef et al., 2011). On the other hand, characterizations of numerous ChR2 variants have focused on step or pulse stimuli (Gunaydin et al., 2010; Mattis et al., 2011). To the best of our knowledge, this study is the first to both theoretically and experimentally derive the ChR2 channel's linear and complete response function in three ChR2 variants.

In this study, we demonstrated the ability of ChR2 to evoke continuously modulated photocurrents in neurons in response to continuously modulated light signals. We used a three-state Markov model (Nagel et al., 2003) to derive an analytical frequency response function for ChR2 variants (Equation 4). We confirmed these model predictions experimentally and have shown that the model is sufficient to capture dynamical properties of ChR2 in neurons within a physiologically relevant frequency range. Additionally, we found that the passband of ChR2, ChR2_R_, and ChR2_A_ are broad enough to support photocurrents that mimic the noisy synaptic input received by neurons in the high conductance state, *in vivo* (Destexhe et al., 2003) (Supplementary Figure S1).

Aside from channel bandwidth, we found that temporal characteristics of continuously-varying photocurrents were highly repeatable across trials and cells. This contrasted with the amplitude distributions of the light-evoked currents across cells, which were highly variable (Figure 4C). The variability of photocurrent amplitudes results from non-homogeneous expression levels across cells. Potentially, this variability in channel expression could be used to simulate natural sources of noise in neural circuits such as variability in the number of incoming projections (Dayan and Abbott, 2001), variable spiking thresholds (Azouz and Gray, 2000), and sodium channel noise (Jacobson et al., 2005).

Continuously-varying optical stimuli allow subthreshold conductance modulations that can be spatially and genetically targeted. The spatially uniform optical stimuli used in our study produced highly correlated photocurrents across cells (Figure 4D). These temporally locked photocurrents mimic the highly correlated state of subthreshold thalamic drive to sensory cortical neurons that share a receptive field (Lampl et al., 1999; Roy and Alloway, 2001; Okun and Lampl, 2008). However, the incorporation of spatial light modulation would open the door to more complex experimental questions. For instance, spatial modulation of continuously-varying stimuli would allow control over the degree of synchronization between subthreshold currents across cells (Reutsky-Gefen et al., 2013). Additionally, spatial light modulation could be used to isolate continuously-varying input to particular regions of individual neurons (Grossman et al., 2010). For instance, by targeting the soma and axon-hillock, any low-pass effect resulting from the integrative properties of the dendritic arbor might be avoided. Conversely, targeting dendrites might provide more biophysically realistic input compared to stimuli covering the entire cell. However, even without spatial control of stimuli, we have shown that spatially homogeneous stimuli provide a wide bandwidth to deliver complex stimulus waveforms to populations of cells. Additionally, spatially homogeneous continuously-varying stimulation has the added benefit that it can be readily incorporated into existing experimental setups that use multi-mode optical fiber to deliver light *in vivo*.

Finally, we showed that ChR2's frequency response function supports a resonance. The degree of resonance is dependent on the values of free model parameters, which change for different ChR2 variants and stimulus signals. This finding is especially relevant for studies that use ChR2 to examine the frequency-dependence of neural circuitry (Cardin et al., 2009), since it is important to separate the intrinsic dynamics of ChR2 from those that belong to the network under study. We found the most pronounced resonance for ChR2 with a natural frequency of ~6–10 Hz. ChR2 was cloned from the green algae *Chlamydomonas reinhardtii*. Interestingly, the algae's phototaxic flagellar movement is tuned to the resonant frequency band of ChR2 (Josef et al., 2006), indicating a potential behavioral significance of ChR2's bandpass effect for algae in their natural environment.

ChR2's amplitude response function indicates that the sum of channel recovery and desensitization transition rates determines its frequency cut-off. Therefore, opsins with faster transition rates will allow a broader passband for time varying inputs. As new optogenetic tools are discovered and existing ones improved, their increased bandwidth may eventually offer an artificial, optical neural communication channel that actually exceeds the bandwidth of natural sensory organs. This would have tremendous implications for how neural computation and processing are studied and for the advancement of brain-machine interfaces. For the purposes of continuously-varying photostimulation with existing tools, we found that the ChR2_A_ variant offered the widest dynamic range, did not display voltage-dependent channel kinetics, and exhibited only a mild resonance. This makes it a good choice for delivering continuously-varying stimuli to populations of cells embedded within functioning neural circuits.

ChR2 was derived from microbes that use it for optical sensation in natural environments. It is therefore not surprising that the channel is excellent at conveying continuously-varying input signals. Using channelrhodopsins as a means for delivering repeatable, continuously-varying stimuli to genetically defined populations of cells will be a powerful method for probing the dynamics of neural circuits and modulating their activity to provide artificial sensation.

## Methods

### Culturing methods

Our culturing methods are described in detail elsewhere (Hales et al., 2010). All experiments were carried out in accordance with the USA Public Health Service's Policy on Humane Care and Use of Laboratory Animals and the Guide for the Care and Use of Laboratory Animals using a protocol approved by the Georgia Tech IACUC. Timed-pregnant female rats were anesthetized with inhaled isoflurane and killed by decapitation. Whole brains were excised from embryonic day 18 (E18) rats. Cortical tissue was digested in a solution of 20 U·ml^−1^ papain (Roche, Penzberg, Germany). Following enzymatic digestion, cells were mechanically dissociated using 3–5 trituration passes through a p1000 pipette tip. The resulting cell suspension was filtered through with a 40 μm cell strainer and then centrifuged at 200·g to remove large and small debris, respectively. The cell pellet was diluted to 2500 cells·μL^−1^. Approximately 50,000 cells in a 20 μL drop were plated at onto a ~2 mm diameter area on glass bottom petri dishes, resulting in ~16,000 cells·mm^2^ on the culturing surface. 0.75 mL of the culturing medium was exchanged every 3 days, for each culture. Cultures dishes were sealed with a Teflon membrane (Potter and DeMarse, 2001) and stored in an incubator regulated to 35°C, 5% CO_2_, 65% relative humidity.

### ChR2 expression system

AAV2-CaMKllα::ChR2-mCherry at 4·10^12^ c.f.u.·ml^−1^ was produced by the Kaplitt lab (Cornell University) using plasmid DNA for CaMKIIα::ChR2-mCherry obtained from the K. Deisseroth (Standford University). AAV2-CaMKllα::hChR2(H134R)-mCherry at 4·10^12^ c.f.u.·ml^−1^ was produced by the University of North Carolina at Chapel Hill Vector Core. AAV9-CaMKllα::hChR2(E123A/H134R)-eYFP at 4·10^12^ c.f.u.·ml^−1^ was produced by the University of Pennsylvania Vector Core. At 1–5 days *in vitro* (DIV), viral aliquots were diluted to 1·10^12^ c.f.u.·ml^−1^ using culturing medium. 1 μL of diluted viral solution was added to 1 mL culturing medium for a final infection concentration of 1·10^9^ c.f.u.·ml^−1^. Cultures were then incubated for 3 days before the culturing medium was exchanged. The fluorescent signal of the reporter protein was monitored for several days post infection to ensure channel expression. All experiments were carried out at 3–4 weeks *in vitro*.

### Intracellular recordings

Whole-cell voltage-clamp recordings were conducted on pyramidal neurons expressing the mCherry (ChR2_R_ and ChR2) or eYFP (ChR2_A_) reporter protein. Recordings were performed in a continuous perfusion of artificial cerebrospinal fluid (aSCF) bubbled with 95% O_2_ and 5% CO_2_ to maintain a pH of 7.4. The aSCF solution contained (in mM) 126 NaCl, 3 KCl, 2 CaCl_2_, 1 NaH_2_PO_4_, 25 NaHCO_3_, 1.5 MgSO_4_, and 25 D-glucose. The temperature of the extracellular medium was regulated to 35°C using an inline heater (Warner Instruments, Hamden, CT). 1.5 mm outer diameter, 1.1 mm inner diameter borosilicate glass capillaries (Sutter Instruments, Novato, CA) were pulled into patch pipettes and filled with a solution containing (in mM) 100 K-gluconate, 30 KCl, 3 ATP, 2 MgSO_4_, 0.5 ethylene glycol tetraacetic acid and 10 HEPES adjusted to pH 7.4 using 0.1 M KOH. Filled pipettes had resistances of 4–8 MΩ. Voltage clamp recordings were performed using HEKA EPC8 amplifier and PatchMaster control software in whole-cell mode. Cells were held at −70 mV and membrane current measurements were amplified and low-pass filtered at 3 kHz before being digitized at 20 kHz and streamed to disk. Access resistance and seal resistance were monitored between stimulation protocols. Current clamp recordings were performed in “fast” mode using the same filter setting as voltage clamp. All experiments were performed in the presence of 40 μM 6-cyano-7-nitroquinoxaline-2,3-dione (CNQX), 50 μM (2R)-amino-5-phosphonovaleric acid (AP5), 20 μM bicuculline in order to prevent synaptic transmission. Whole-cell recordings were analyzed offline in MATLAB (The MathWorks, Natick, MA).

### Optical stimulation

A 10-watt (electrical power) light emitting diode (LED) was used for optical stimulation, with peak emission wavelength of 465 nm and ~20 nm full width at half maximum intensity (LZ4-00B200, LEDEngin, San Jose, CA). To deliver optical stimuli to cultured neurons, the LED was focused into the epi-illumination port of an E600FN upright microscope (Nikon Corporation, Tokyo, Japan) and passed through a 40X objective lens. The light power produced by LEDs is affected by their temperature. Additionally, the relationship between forward diode current and irradiance is a static non-linearity. To compensate for these factors and deliver distortion-free optical stimuli, we designed a precision current source with integrated optical-feedback to drive our LED (Figure 3A). This circuit measures the instantaneous optical power produced by the LED using an amplified photodiode. It then adjusts the current sourced to the LED such that the optical power measurement matches a reference voltage supplied by a digital to analog converter (DAC; LIH 1600, HEKA Electronik, Lambrecht/Pfalz, Germany). The circuit can precisely modulate the LED brightness over a bandwidth of 50 kHz (Figures 3B,C). A full design specification for the device is available online (https://potterlab.gatech.edu/newman/wiki).

### Derivation of ChR2's frequency response

The differential equations governing the Markov model, (Equations 1–3), are non-homogeneous with continuously-varying coefficients. For this reason, the frequency response function does not provide a full description of the model's dynamics. However, it serves as a useful simplification for describing ChR2's bandpass characteristics within local regions of optical intensity (Figure 1B). The full time-variant dynamics are not analytically solvable and required numerical simulations of response trajectories (Figure 2).

ChR2's frequency response function, *F*_ChR2_(ω), can be obtained by considering the channels' response to a small sinusoidal light signal with a constant light level ϕ_0_,

(8) $$\phi (t)=\phi_{0}+\delta \phi \exp(j\omega t),$$

where ω= 2π*f* and *f* is the frequency of the sinusoid in Hz and $j=\sqrt{-1}$. The first order response dynamics of the open and closed states can then be described by a constant offset and periodic component,

(9) $$O(t)=O_{0}+\delta O\exp(j\omega t)$$

(10) $$D(t)=D_{0}+\delta D\exp(j\omega t).$$

Within a local region of optical intensities, ϕ_0_ ± δϕ, changes in the open state, δ*O*, or the desensitized state, δ*D*, are proportional to changes in optical input, ϵδϕ. The proportionality factors for the open and desensitized states are the frequency response functions *F*_ChR2_(ω) and *G*_ChR2_(ω), respectively,

(11) $$\delta O=\epsilon \delta \phi F_{\text{ChR2}}(\omega )$$

(12) $$\delta D=\epsilon \delta \phi G_{\text{ChR2}}(\omega ).$$

Differentiating Equations 9 and 10 and inserting the result into Equations 1 and 2 leads to 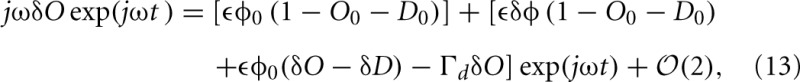

(14) $$j\omega \delta D\exp(j\omega t)\text{​}=\text{​​}\left[\Gamma_{d}O_{0}-\Gamma_{r}D_{0}\right]+\text{​}\left[\Gamma_{d}\delta O-\Gamma_{r}\delta D\right]\exp(j\omega t)\text{​}.$$

By dropping all but the first-order terms of Equations 13 and 14 (meaning those terms proportional to exp(*j*ω*t*)), and removing the common factor exp(*j*ω*t*), changes in the open and desensitized states due to changes in light power are given by

(15) $$j\omega \delta O=\epsilon \delta \phi \text{​}\left(1-O_{0}-D_{0}\right)+\text{​}\left(\epsilon \phi_{0}-\Gamma_{d}\right)\delta O-\epsilon \phi_{0}\delta D$$

(16) $$j\omega \delta D=\Gamma_{d}\delta O-\Gamma_{r}\delta D,$$

where (1 − *O*_0_ − *D*_0_) = *C*_0_ is the steady-state probability of the channel being closed. Performing the necessary algebra to solve for δ*O* results in

(17) $$\delta O=\epsilon \delta \phi \text{​}\left[\frac{C_{0}(j\omega +\Gamma_{r})}{-\omega^{2}+j\omega \text{​}\left(\Gamma_{r}+\epsilon \phi_{0}+\Gamma d\right)+\epsilon \phi_{0}\Gamma_{r}+\epsilon \phi_{0}\Gamma_{d}+\Gamma_{r}\Gamma_{d}}\right].$$

Finally, referencing Equation 11, ChR2's frequency response function for a local region of light intensities is calculated by dividing the left hand side of Equation 17 by ϵδϕ,

(18) $$\begin{array}{l} F_{\text{ChR2}}(\omega )=\frac{\delta O}{\epsilon \delta \phi }\\ \;=\frac{C_{0}(j\omega +\Gamma_{r})}{-\omega^{2}+j\omega (\Gamma_{r}+\epsilon \phi_{0}+\Gamma_{d})+\epsilon \phi_{0}\Gamma_{r}+\epsilon \phi_{0}\Gamma_{d}+\Gamma_{r}\Gamma_{d}} \end{array}$$

and the amplitude response is then given by

(19) $$|F_{\text{ChR2}}|=\frac{C_{0}\sqrt{\omega^{2}+\Gamma_{r}^{2}}}{\sqrt{{\left(-\omega^{2}+\epsilon \phi_{0}\Gamma_{r}+\epsilon \phi_{0}\Gamma_{d}+\Gamma_{r}\Gamma_{d}\right)}^{2}+{\left(\omega \text{​}\left(\Gamma_{r}+\epsilon \phi_{0}+\Gamma_{d}\right)\right)}^{2}}}.$$

In the high frequency limit, Equation 4 reduces to $\frac{C_{0}(j\omega )}{-\omega^{2}+j\omega (\Gamma_{r}+\epsilon \phi_{0}+\Gamma_{d})}\propto \frac{C_{0}}{j\omega /(\Gamma_{r}+\epsilon \phi_{0}+\Gamma_{d})+1}$.

### Experimental verification of frequency response functions

To estimate the frequency response of ChR2, $\hat{F}$_ChR2_(ω), we delivered optical stimuli, *s*(*t*), consisting of *T* = 10 s realizations of a Gaussian (Ornstein-Uhlenbeck) noise process while recording evoked intracellular currents, *I*_*i*_(*t*), within a single cell, *i*. *s*(*t*) was generated according to

(20) $$s(t_{n+1})=\mu_{s}+s(t_{n})\exp\text{​}\left(-dt/\tau_{s}\right)+\sigma_{s}\xi (t_{n})\sqrt{1-\exp(-2dt/\tau_{s})},\;$$

where *s*(*t*_1_) = 0 mW·mm^−2^, μ_*s*_ ≃ 0.35 mW·mm^−2^, and σ_*s*_ ≃ 0.08 mW·mm^−2^ are the initial condition, mean, and standard deviation of the process, respectively. τ_*s*_ = 50 ms is the correlation time of *s*(*t*), *dt* = 40 μs is the DAC update period, and ξ(*t*_*n*_) is a random variable drawn from the standard normal distribution. Each cell was exposed to a single, repeated realization of *s*(*t*) for *k* = 10 trials. The first 500 ms of each trial was ignored to remove the non-stationary effects of the stimulator turning on. The recorded intracellular currents were averaged across trials,

(21) $$\langle I_{i}\rangle =\frac{1}{10}\sum_{k=1}^{10}I_{i,k}(t)$$

to remove trial-to-trial noise. We then calculated the empirical frequency response function for each cell,

(22) $${\hat{F}}_{\text{ChR2},i}(\omega )=\frac{S_{s\langle I_{i}\rangle }}{S_{ss}},$$

where *S*_*ss*_ is the power spectrum of *s*(*t*) and *S*_*s*〈*I*_*i*_〉_ is the cross spectrum of 〈*I*_*i*_〉 and *s*(*t*). *S*_*ss*_ and *S*_*s*〈*I*_*i*_〉_ are defined as the Fourier transforms of the corresponding correlation function,

(23) $$c_{ss}(\tau )=\int_{-T}^{T}\text{​}s(t)s(t+\tau )\text{d}\tau$$

(24) $$c_{s\langle I_{i}\rangle }(\tau )=\int_{-T}^{T}\text{​}s(t)I(t+\tau )\text{d}\tau .$$

Finally, we averaged $\hat{F}$_ChR2, *i*_(ω) across cells to obtain the empirical frequency response for each construct, $\hat{F}$_ChR2_(ω). To improve our estimate of the power spectra, we followed the procedure introduced in Higgs and Spain (2009) and used a frequency dependent window, equivalent to a Gaussian bandpass filter with standard deviation of σ = 2π/ω in the frequency domain. Spectra were evaluated at discrete increments, ω_*n*_ = 2π10^*n*^, *n* = 0.1, 0.2, …, 3. Model parameters were obtained for each ChR2 variant by fitting the predicted frequency response function in Equation 4 to the experimental estimate, Equation 22.

In addition to Gaussian stimuli, we used cosine frequency sweeps (“chirps”) consisting of *T* = 20 s sinusoidal sweeps of constant amplitude *a*_*s*_ and exponentially increasing frequency from *f*_0_ = 0.1 to *f*_*T*_ = 1000 Hz. They were defined as

(25) $$s(t)=a_{s}\cos(2\pi f(t))+a_{0}$$

where

(26) $$f(t)=f_{0}{\left(f_{T}/f_{0}\right)}^{\frac{t}{T}}$$

and *a*_*s*_ ≃ 0.3 mW·mm^−2^ and *a*_0_ ≃ 0.35 mW·mm^−2^. The empirical frequency response was then estimated directly from the intracellular current recordings according to 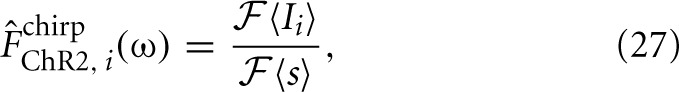 where 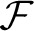 denotes the Fourier transform.

### Conflict of interest statement

The authors declare that the research was conducted in the absence of any commercial or financial relationships that could be construed as a potential conflict of interest.
