# Supplementary material for: Delivery of continuously-varying stimuli using channelrhodopsin-2
Source: Front Neural Circuits. 2013 Dec 6;7:184. doi: 10.3389/fncir.2013.00184 (PMC3853882; doi:10.3389/fncir.2013.00184)
Supplement: Supplementary file 1 [file SupplementaryMaterial.PDF]

Supplementary Material: Delivery of continuously-varying stimuli using channelrhodopsin-2

Tatjana Tchumatchenko[1,2,*], Jonathan P. Newman[3,*], Ming-fai Fong[3,4], Steve M. Potter[3]

[1] Center for theoretical Neuroscience,
Columbia University College of Physicians and Surgeons,
1051 Riverside Dr, New York, NY 10032, USA
[2] Max Planck Institute for Brain Research,
Deutschordenstr. 46, 60528 Frankfurt am Main, Germany
[3] Dept. of Biomedical Engineering, Georgia Institute of Technology, 313 Ferst Drive, Atlanta, GA, 30332, USA
[4] Dept. of Physiology, Emory University School of Medicine, 201 Dowman Drive. Atlanta, GA 30322, USA
∗ Co-first authors; These authors contributed equally.

Date: December 2, 2013

List of Figures

S1 Example membrane voltages during Gaussian photostimululation 2
S2 Empirical amplitude response functions derived from chirp stimuli. 3
S3 Amplitude distributions for chirp and Ornstein-Uhlenbeck stimuli 4
S4 Time-invariant versus complete ChR2 responses to 20 Hz stimuli 5

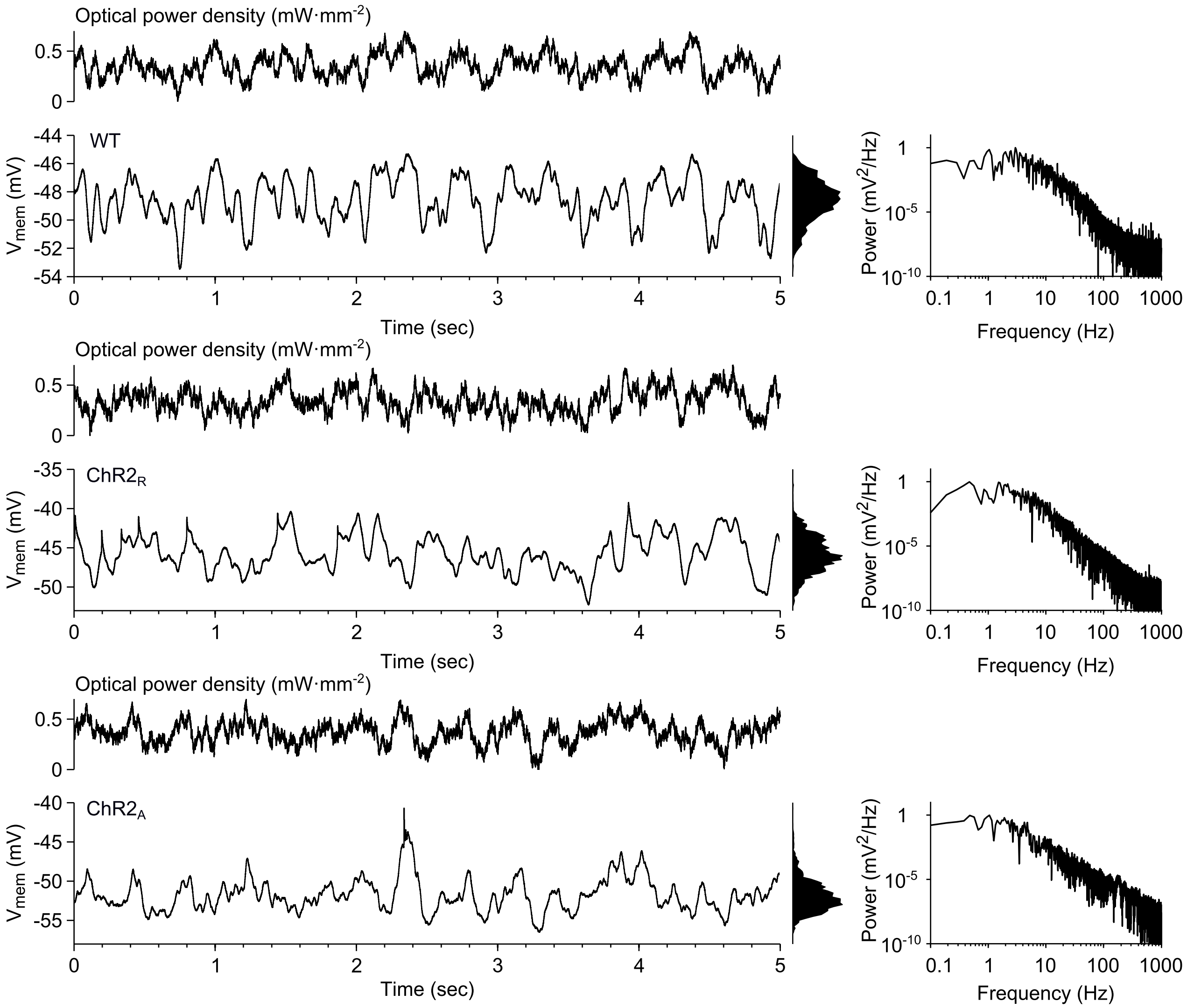

Figure S1: Example membrane voltages during Gaussian photostimulation. (Left) Evoked voltage fluctuations obtained from current-clamp recordings performed during time varying photo-stimulation are shown for each ChR2 variant used in this study. Each trace is the average of 10 stimulus-response pairs to a repeated Ornstein-Uhlenbeck stimulus waveform. A voltage histogram is shown to the right of each trace. (Right) Power spectral density estimates for the voltage response. Compare this figure to box 1 in reference [S1].

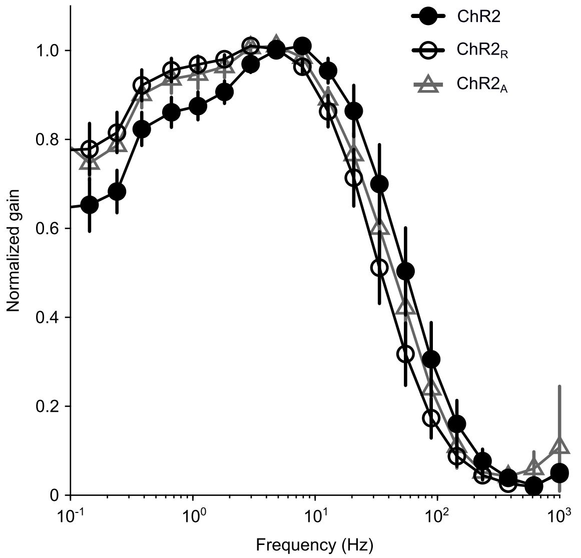

Figure S2: Empirical amplitude response functions of wtChR2 and engineered variants derived from chirp stimuli.

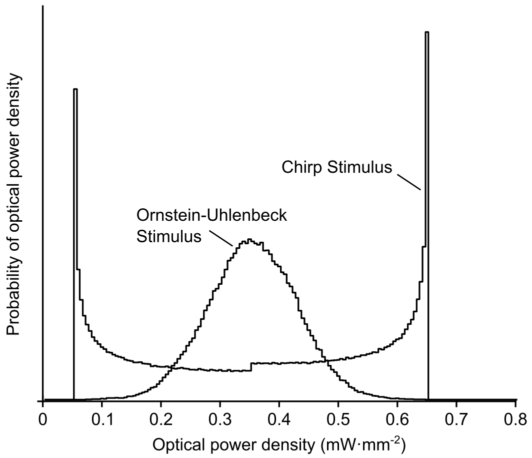

Figure S3: Light power distributions for chirp and Ornstein-Uhlenbeck stimuli.

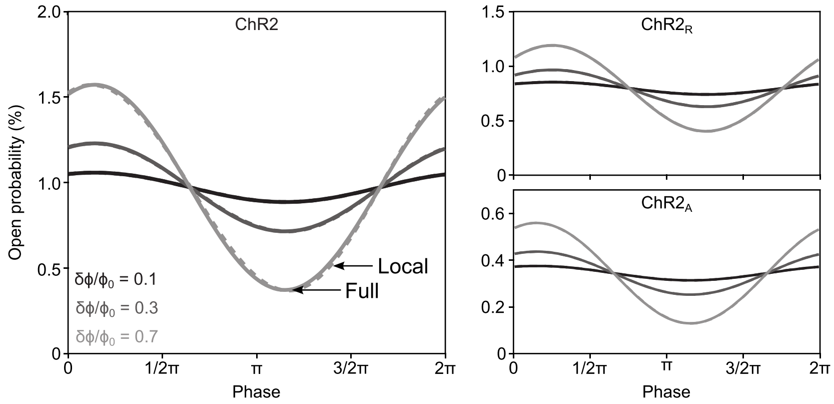

Figure S4: Time-invariant versus complete model dynamics for different ChR2 variants for 20 Hz stimuli. The time-invariant approximation (solid lines) and the complete linear response (dashed lines) of $O(t)$ are shown in response to 20 Hz sinusoidal stimuli for ChR2 (left), ChR2$_{\mathrm{R}}$ (right, top) and ChR2$_{\mathrm{A}}$ (right, bottom). Gray shades represent different sinusoidal amplitudes normalized to the mean stimulus intensity, $\delta\phi/\phi_0 =$ 0.1, 0.3, and 0.7.

References

[S1] Destexhe A, Rudolph M, Pare D (2003) The high-conductance state of neocortical neurons *in vivo*. Nat Rev Neurosci 4: 739-751.
